# Population modeling of bosutinib exposure‐response in patients with newly diagnosed chronic phase chronic myeloid leukemia

**DOI:** 10.1002/cam4.6439

**Published:** 2023-08-08

**Authors:** May Garrett, Beverly Knight, Jorge E. Cortes, Michael W. Deininger

**Affiliations:** ^1^ Pfizer Oncology San Diego California USA; ^2^ Georgia Cancer Center Augusta Georgia USA; ^3^ Versiti Blood Research Institute Milwaukee Wisconsin USA

**Keywords:** bosutinib, chronic myeloid leukemia, efficacy, PK/PD, safety

## Abstract

**Background:**

The BELA and BFORE trials compared bosutinib starting doses of 500 mg once daily (QD) and 400 mg QD, respectively, with imatinib in adults with newly diagnosed chronic phase chronic myeloid leukemia (CP‐CML). The B1871048 trial evaluated bosutinib 400 mg QD in Japanese patients with newly diagnosed CP‐CML.

**Aim:**

This analysis assessed the impact of a lower bosutinib starting dose on key efficacy and safety outcomes.

**Materials & Methods:**

A pharmacokinetic model was used to estimate metrics of bosutinib exposure, and logistic regression was used to investigate relationships with efficacy (cumulative major molecular response [MMR] and cumulative complete cytogenetic response [CCyR]) and safety outcomes (eight prespecified adverse events).

**Results:**

Totals of 573 and 574 patients were included in the efficacy and safety endpoint analyses, respectively. Cumulative MMR and CCyR were similar across studies. Log(*C*_trough_) and log(*C*_avg_) were significant predictors of MMR and CCyR, and the probability of achieving MMR or CCyR increased 1.3‐fold or 2.7‐fold for every 1 unit increase in log(*C*_trough_) or log(*C*_avg_), respectively. An exposure–response relationship was identified between time‐to‐event and risk of diarrhea, nausea, and vomiting. Significant relationships were also observed between time‐to‐event and log(*C*_avg_), *C*_trough_, and *C*_avg_ with diarrhea, nausea, and vomiting, respectively.

**Discussion:**

A bosutinib exposure‐response relationship with safety and efficacy was observed.

**Conclusion:**

Compared with 500 mg QD, a bosutinib starting dose of 400 mg QD improved tolerability in some patients with newly diagnosed CP‐CML without compromising efficacy. ClinicalTrials.gov identifiers: NCT00574873; NCT02130557; NCT03128411.

## 1 INTRODUCTION

Chronic myeloid leukemia (CML) is characterized by a chromosomal translocation leading to the generation of the Philadelphia (Ph) chromosome and expression of the *BCR*::*ABL1* fusion protein. Beginning with the approval of imatinib in 2001, tyrosine kinase inhibitors (TKIs) targeting *BCR*::*ABL1* have transformed the therapeutic landscape for CML, for most patients turning a lethal disease into a chronic condition.

Bosutinib is an orally active, dual Src/Abl TKI that displays greater potency against *BCR*::*ABL1* when compared with imatinib.^1^, ^2^ In clinical trials, bosutinib has demonstrated clinical efficacy and a manageable safety profile in adult patients with chronic phase CML (CP‐CML) resistant or intolerant to prior therapy.^3^, ^4^, ^5^ The approved dose of bosutinib in this setting is 500 mg once daily (QD),^6^ which was selected for further assessment following a phase 1/2 study that did not identify a maximum tolerated dose and reported efficacy and early safety signals that did not meet the criteria for dose‐limiting toxicity.^3^ The Phase 3 BELA trial compared bosutinib 500 mg QD with imatinib 400 mg QD in adult patients with newly diagnosed CP‐CML. This trial failed to meet its primary efficacy endpoint, which was complete cytogenetic response (CCyR) rate at 12 months, possibly owing to early bosutinib discontinuations due to adverse events (AEs).^7^ This finding was based on an intent‐to‐treat (ITT) analysis, yet many patients did not have cytogenetic data and, hence, were considered failures for the purpose of ITT assessment. However, bosutinib was superior to imatinib in the key secondary endpoint—major molecular response (MMR) rate at 12 months. The subsequent Phase 3 BFORE trial compared a reduced bosutinib starting dose of 400 mg QD with imatinib in adult patients with newly diagnosed CP‐CML and showed that the MMR rate at 12 months (the primary endpoint) and cumulative CCyR rate at 12 months were both significantly higher in the modified ITT population randomized to bosutinib (Ph‐positive patients with e13a2/e14a2 transcripts).^8^ Based on the results from BFORE, a bosutinib starting dose of 400 mg QD was subsequently approved for use in the first‐line setting in December 2017.^6^ B1871048 was a Phase 2, single‐arm, open‐label trial designed to evaluate the efficacy and safety of 400 mg QD bosutinib monotherapy in 60 adult Japanese patients with newly diagnosed CP‐CML.^9^ The primary objective of the study (MMR at Month 12) was met, with no on‐treatment transformations to accelerated/blast phase. No patient died on treatment or within 28 days of the last bosutinib dose, and there were no new safety signals for bosutinib. These data support the efficacy and safety of 400 mg QD bosutinib monotherapy in Japanese patients with newly diagnosed CP‐CML.^9^

The present analysis integrated data from the BELA, BFORE and B1871048 trials in order to assess quantitatively the impact of reducing the starting dose of bosutinib from 500 to 400 mg QD with respect to the relationships between pharmacokinetic (PK) metrics of bosutinib exposure and key efficacy and safety outcomes in adult patients with newly diagnosed CP‐CML. It also aimed to identify potential covariates that may be important predictors of variability in bosutinib distribution and elimination.

## 2 METHODS

### 2.1 Study designs of BELA, BFORE, and B1871048

The BELA (NCT00574873) and BFORE (NCT02130557) trials were randomized, open‐label, Phase 3 studies of first‐line bosutinib versus imatinib in CP‐CML.^7^, ^8^ The B1871048 (NCT03128411) trial was an open‐label, Phase 2 study of first‐line bosutinib in CP‐CML in newly diagnosed Japanese patients.^9^ In both BELA and BFORE, eligible participants were ≥ 18 years of age, while they were ≥ 20 years of age for B1871048. Patients in all three studies had a recent diagnosis of CP‐CML (≤6 months from initial diagnosis), an Eastern Cooperative Oncology Group performance status of 0 or 1, and no prior treatment for CML (including TKIs). Oral bosutinib was administered at a starting dose of 500 mg QD in BELA and 400 mg QD in BFORE and B1871048, with dose escalation up to a maximum of 600 mg QD permitted in patients with a suboptimal response and no Grade 3/4 AEs or persistent Grade 2 AEs. Dose reduction to 300 mg QD was allowed for prespecified treatment‐related toxicities (and down to a minimum of 200 mg QD in B1871048 with specific sponsor approval).

### 2.2 Bosutinib exposure metrics

Individual bosutinib plasma exposure estimates were used to assess potential exposure‐response relationships. Bosutinib exposure metrics were calculated from a population PK model, which was a two‐compartment model with first‐order absorption and an absorption lag time. The PK model was based on pooled data from 10 clinical studies: a Phase 1 study of bosutinib in advanced solid tumors (NCT00195260)^10^; a phase 1/2 study of bosutinib in Ph‐positive leukemias (NCT00261846)^3^, ^4^, ^5^; three Phase 1 studies involving healthy adult patients (B1871021 [NCT00406406], B1871035 [NCT01374139], B1871044 [NCT02192294]); one Phase 1 study involving patients with hepatic impairment and matched healthy adult patients (B1871003 [NCT00759837]); one Phase 1 study involving patients with renal impairment and matched healthy adult patients (B1871020 [NCT01233882]); and the three studies previously described involving patients with CML (BELA,^7^ BFORE,^8^ B1871048^9^). For some studies included in the PK model, only subsets of clinically relevant data were used in the PK analysis. Studies varied in terms of bosutinib formulation, route of administration (study B1871044 included intravenous and oral administration), fed versus fasted state, and patient population.

### 2.3 PK sampling

Blood samples were collected and used to characterize the PK profile of bosutinib in the population PK model. For the majority of the studies, extensive PK sampling was conducted; however, in the studies involving patients with CML, only sparse sampling was conducted. In BELA, eight blood samples per patient were collected: predose and 3 and 6 h postdose on Days 1 and 28, and predose on Days 56 and 84. In BFORE and B1871048, four blood samples per patient were collected: predose on Days 1, 28, 56, and 84.

### 2.4 Exposure–efficacy analysis

Patients with at least one postdose value and the appropriate PK exposure information for bosutinib were included in the exposure‐response analysis of key efficacy outcomes, which were cumulative MMR and cumulative CCyR (any on‐treatment response). Exposure‐response analysis was performed using binominal logistic regression to assess the relationships between key efficacy outcomes and specific metrics of bosutinib exposure (linear and log‐transformed) based on lowest deviance value (cumulative area under the concentration–time curve up to time to initial response [cAUC], average concentration calculated as the ratio of cAUC over the respective timeframe [*C*_avg_], and trough concentration prior to initial response [*C*_trough_]; the same three metrics were calculated at Day 28 of treatment [cAUC28, *C*_avg28_, and *C*_trough28_]). Demographic factors and baseline safety laboratory values were tested for significance on response using a backward elimination algorithm with a cutoff of *p* < 0.01.

### 2.5 Exposure–safety analysis

In the exposure‐response analysis of key safety outcomes, the risks of eight prespecified AEs selected for their clinical relevance were investigated (diarrhea, nausea, vomiting, rash, elevated alanine aminotransferase [ALT], elevated aspartate aminotransferase [AST], thrombocytopenia, and neutropenia). AEs were captured as ordered events graded on a 5‐point scale (0–4), with 0 representing no AE and 4 representing the most severe grade of AE. AEs were based on the National Cancer Institute's Common Terminology Criteria for Adverse Events (NCI CTCAE), using the scale applicable at the time of each study. No Grade 5 AEs were observed in any of the studies. Only the first occurrence of the highest‐grade AE within the first year of bosutinib treatment was included in the analysis. The analysis did not account for patients who dropped out. For each of the eight prespecified AEs, bosutinib *C*_avg_ and *C*_trough_ prior to the event were evaluated with a time‐to‐event parameter using ordinal logistic regression. If ordinal logistic regression showed no statistically significant relationship for any of the safety endpoints, additional analyses were performed with binary logistic regression. Time‐to‐event (the day prior to the occurrence of the highest‐grade AE divided by the 366 days in 1 year) was tested in the base model with each bosutinib exposure metric. If none of bosutinib exposures were found to be statistically significant when incorporating the time‐to‐event parameter, then the AE was re‐evaluated without this parameter in the model. If a patient had no AE (Grade 0), then time‐to‐event was set to the 1‐year landmark (366/366 = 1). *p* < 0.05 was considered statistically significant. An appropriate baseline laboratory parameter was included in the base model to account for any influences on the model for elevated ALT, elevated AST, and thrombocytopenia. In general, each patient had one record for each endpoint in the analyses of potential correlations between bosutinib exposure and safety outcomes.

## 3 RESULTS

### 3.1 Patients and metrics of bosutinib exposure

Based on appropriate PK and efficacy data availability, a total of 573 patients (from studies BELA, BFORE, B1871048) were included in the exposure–response analysis for MMR; for CCyR, the exposure–response analysis was conducted in Ph + patients only (*N* = 554). A total of 574 patients were included in the exposure–response analysis for key safety outcomes.

Patient demographics and baseline laboratory values in the bosutinib arms of BELA, BFORE, and B1871048 are shown in Table 1. There was a higher proportion of White patients (78.9% vs 64.0%) in BFORE versus BELA, and a lower proportion of Asian patients (12.0% vs. 26.3%) in BFORE versus BELA; B1871048 enrolled Japanese patients only. Patients in B1871048 had a smaller body surface area than the other two studies. In addition, there was a greater proportion of patients with ECOG PS 0 in B1871048 compared with BELA and BFORE (96.7% vs. 74.6% and 72.9%, respectively). Median baseline laboratory values were similar across all three studies, with the exception that patients in B1871048 had higher alkaline phosphatase (AP) and platelets compared to BELA and BFORE. In addition, patients in BFORE had a higher median baseline absolute neutrophil count compared to patients in BELA and B1871048.

**TABLE 1:** Summary of baseline categorical and continuous covariates.

Variable	BELA (*N* = 247)	BFORE (*N* = 266)	B1871048 (*N* = 60)
Age, median (range), years	48.0 (19–91)	53.0 (18–84)	55.0 (20–83)
Sex, *n* (%)			
Male	148 (59.9)	156 (58.6)	36 (60.0)
Female	99 (40.1)	110 (41.4)	24 (40.0)
Race, *n* (%)			
White	158 (64.0)	210 (78.9)	0
Asian	65 (26.3)	32 (12.0)	60 (100)
Black	2 (0.8)	10 (3.8)	0
Other	22 (8.9)	14 (5.3)	0
ECOG PS, *n* (%)			
0	184 (74.5)	194 (72.9)	58 (96.7)
1	63 (25.5)	72 (27.1)	2 (3.3)
Body weight, median (range), kg	*N* = 247 69.0 (35.0–136.0)	*N* = 264 75.6 (35.0–125.0)	*N* = 60 59.8 (33.2–102.8)
CCL, median (range), mL/min	*N* = 246 97.7 (40.0–249.0)	*N* = 264 102.3 (32.5–250.5)	*N* = 60 101.0 (36.9–196.8)
AP, median (range), U/L	*N* = 245 89.0 (30.5–792.0)	*N* = 263 78.0 (28.0–508.0)	*N* = 60 206.5 (99.0–791.0)
Albumin, median (range), g/dL	*N* = 243 4.4 (3.3–7.4)	*N* = 260 4.3 (1.9–5.4)	*N* = 60 4.5 (3.7–5.4)
ALT, median (range), U/L	*N* = 246 23.0 (6.0–125.0)	*N* = 265 21.0 (5.0–91.0)	*N* = 60 25.0 (4.0–83.0)
AST, median (range), U/L	*N* = 246 24.0 (8.7–62.0)	*N* = 262 24.0 (5.0–76.0)	*N* = 60 26.0 (13.0–61.0)
Bilirubin, median (range), mg/dL	*N* = 244 0.5 (0.1–4.4)	*N* = 265 0.5 (0.2–1.9)	*N* = 60 0.6 (0.3–1.3)
ANC, median (range), ×10^9^ cells/L	*N* = 230 13.1 (0–202.8)	*N* = 263 26.2 (0.4–342.7)	*N* = 60 18.7 (1.8–123.5)
Platelets, median (range), ×10^9^ cells/L	*N* = 247 386.0 (60.0–4189.0)	*N* = 266 385.5 (72.0–2195.0)	*N* = 60 458.0 (141.0–2133.0)

Abbreviations: ALT, alanine aminotransferase; ANC, absolute neutrophil count; AP, alkaline phosphatase; AST, aspartate aminotransferase; CCL, creatinine clearance; ECOG PS, Eastern Cooperative Oncology Group Performance Status; NCI, National Cancer Institute Organ Dysfunction Working Group.

Mean and median values for the derived exposure metrics by study are shown in Table S1. Overall, the predicted bosutinib exposures prior to initial responses (MMR or CCyR) and at Day 28 were higher in BELA (starting dose of 500 mg QD) compared to BFORE and B1871048 (both with a starting dose of 400 mg QD).

### 3.2 Predictors of variability in bosutinib distribution and elimination from the PK Model

Bosutinib systemic clearance (CL) was estimated to be 56.3 L/h with 43.4% inter‐individual variance (IIV). Central volume of distribution (V_2_), the hypothetical volume when the drug is initially distributed upon administration, was estimated to be 1325.7 L with 38.1% IIV. First‐order absorption rate constant (k_a_) was estimated to be 0.358 per hour with 112.1% IIV. The lag time from dose administration to the beginning of the first‐order absorption was estimated to be 0.442 h, and absolute bioavailability (F) was estimated to be 33.1%. The residual variability was 48.1% and 39.9% for intravenous and oral administration, respectively.

Demographic factors and measures of renal and hepatic function were evaluated as possible covariates contributing to the variability in PK parameters of bosutinib (Table 1). Of those covariates tested, Asian race and baseline creatinine clearance were statistically significant (*α* = 0.001) on systemic CL; age, baseline body weight and Asian population were statistically significant on V_2_. None of the other studied covariates were found to significantly affect the PK of bosutinib. For younger patients aged 29 years (10th percentile), V_2_ lowered by 9.6% relative to V_2_ for a median age of 51 years. Conversely, for older patients aged 69 years (90th percentile), V_2_ was higher by 5.5% relative to V_2_ for a median age of 51 years. Body weight correlated with an increase in bosutinib V_2_. Relative to V_2_ for a median body weight of 72.5 kg, V_2_ lowered by 12.9% for patients with a low body weight of 55 kg (10th percentile). Conversely, for patients with a high body weight of 100 kg (90th percentile), V_2_ was higher by 17.4%. The Asian population had a decrease of 18.4% (95% CI: 13.7%, 23.2%) and 20.2% (95% CI: 12.7%, 27.8%) in CL and V_2_, respectively, relative to the non‐Asian population.

When assessing the effect of renal function upon the PK of bosutinib based on baseline creatinine clearance (BCCL), it was found that, relative to an individual with normal renal function (BCCL = 98.6 mL/min), bosutinib CL decreased by 5.3% for mild renal impairment (BCCL = 75 mL/min), 14.4% for moderate renal impairment (BCCL = 45 mL/min), 25.7% for severe renal impairment (BCCL = 22 mL/min), and 31.1% for end‐stage renal disease (BCCL = 15 mL/min). There were no discernible changes in bosutinib PK with baseline National Cancer Institute Organ Dysfunction Working Group (NCI ODWG) criteria for hepatic impairment or with any of the baseline laboratory values. Additionally, baseline AST and baseline total bilirubin were not significant covariates on bosutinib CL.

### 3.3 Exposure–response analysis for key efficacy endpoints

Overall, the observed responses for cumulative MMR and cumulative CCyR were similar across the three studies evaluated in the exposure‐response analysis. MMR was achieved in 68% of patients in BELA, 74% in BFORE and 70% in B1871048. CCyR was achieved in 80% of patients in BELA and B1871048, and 83% in BFORE. Using pooled data across all three studies, the predicted bosutinib exposures were higher in patients with an MMR or CCyR response compared to those without a response (Table 2).

**TABLE 2:** Metrics of bosutinib exposure in PK‐evaluable patients by MMR and CCyR response and non‐response.

Parameter	Patients with response	Patients without response
Endpoint: MMR^a^	*N* = 407	*N* = 166
cAUC, μg h/mL		
Median (range)	595.7 (148.5–5959.4)	247.1 (12.1–9236.1)
Mean (SD)	943.8 (917.4)	845.5 (1433.1)
*C*_avg_, ng/mL		
Median (range)	102.0 (26.6–296.0)	71.7 (4.4–291.9)
Mean (SD)	108.6 (40.5)	77.1 (40.5)
*C*_trough_, ng/mL		
Median (range)	75.7 (5.2–220.5)	47.0 (0.01–299.3)
Mean (SD)	80.3 (33.5)	53.8 (45.5)
cAUC_28_, μg h/mL		
Median (range)	60.8 (17.5–173.4)	56.3 (9.3–172.0)
Mean (SD)	65.4 (24.9)	58.5 (26.6)
*C*_avg28_, ng/mL		
Median (range)	93.9 (27.0–267.5)	86.9 (14.4–265.4)
Mean (SD)	100.9 (38.4)	90.3 (41.0)
*C*_trough28_, ng/mL		
Median (range)	75.5 (2.9–222.4)	69.7 (0.4–247.5)
Mean (SD)	81.2 (36.5)	72.3 (39.8)
Endpoint: CCyR (Ph + patients only)^b^	*N* = 450	*N* = 104
cAUC, μg h/mL		
Median (range)	279.5 (42.1–2040.5)	143.3 (15.7–4671.6)
Mean (SD)	348.0 (233.0)	248.7 (485.4)
*C*_avg_, ng/mL		
Median (range)	98.0 (20.6–279.6)	58.8 (4.4–196.0)
Mean (SD)	104.0 (39.9)	66.1 (31.4)
*C*_trough_, ng/mL		
Median (range)	72.2 (0.1–249.4)	53.9 (0.01–231.9)
Mean (SD)	79.0 (34.9)	55.2 (42.1)
cAUC28, μg h/mL		
Median (range)	60.9 (17.5–173.4)	53.0 (9.3–172.0)
Mean (SD)	65.3 (25.0)	56.9 (27.5)
*C*_avg28_, ng/mL		
Median (range)	94.0 (27.0–267.5)	81.8 (14.4–265.4)
Mean (SD)	100.7 (38.6)	87.8 (42.4)
*C*_trough28_, ng/mL		
Median (range)	75.2 (2.9–247.5)	69.2 (0.4–231.5)
Mean (SD)	80.8 (37.1)	70.6 (40.6)

Abbreviations: AUC, area under the concentration‐time curve; AUC_28_, cumulative AUC up to Day 28; cAUC, cumulative AUC up to time to initial response; *C*_avg:_ average concentration calculated as the ratio of cAUC over the respective time frame; *C*_avg28_, average concentration calculated as the ratio of cAUC28 over the respective time frame; CCyR, complete cytogenetic response; *C*_trough_, trough concentration at time of initial response; *C*_trough28_, trough concentration on Day 28; MMR, major molecular response.

^a^ A total of 573 patients were included in the exposure–response analysis of cumulative MMR.

^b^ Only Ph+ patients were included in the analysis of cumulative CCyR (*N* = 554).

A significant exposure–response relationship was identified between bosutinib exposures of log(*C*_trough_) and log(*C*_avg_) with cumulative MMR and cumulative CCyR, respectively; none of the demographic covariates or baseline safety laboratory measures were identified as significant predictors in the final models. The probability of achieving MMR increased 1.3‐fold for every 1 unit increase in log(*C*_trough_), and the probability of achieving CCyR increased 2.7‐fold for every 1 unit increase in log(*C*_avg_). Final model parameter estimates are shown in Table S2 and final logistic regression equations are presented below.

Final logistic regression equation for cumulative MMR:

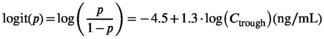




Final logistic regression equation for cumulative CCyR:

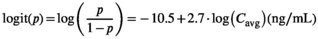




**FIGURE 1:**
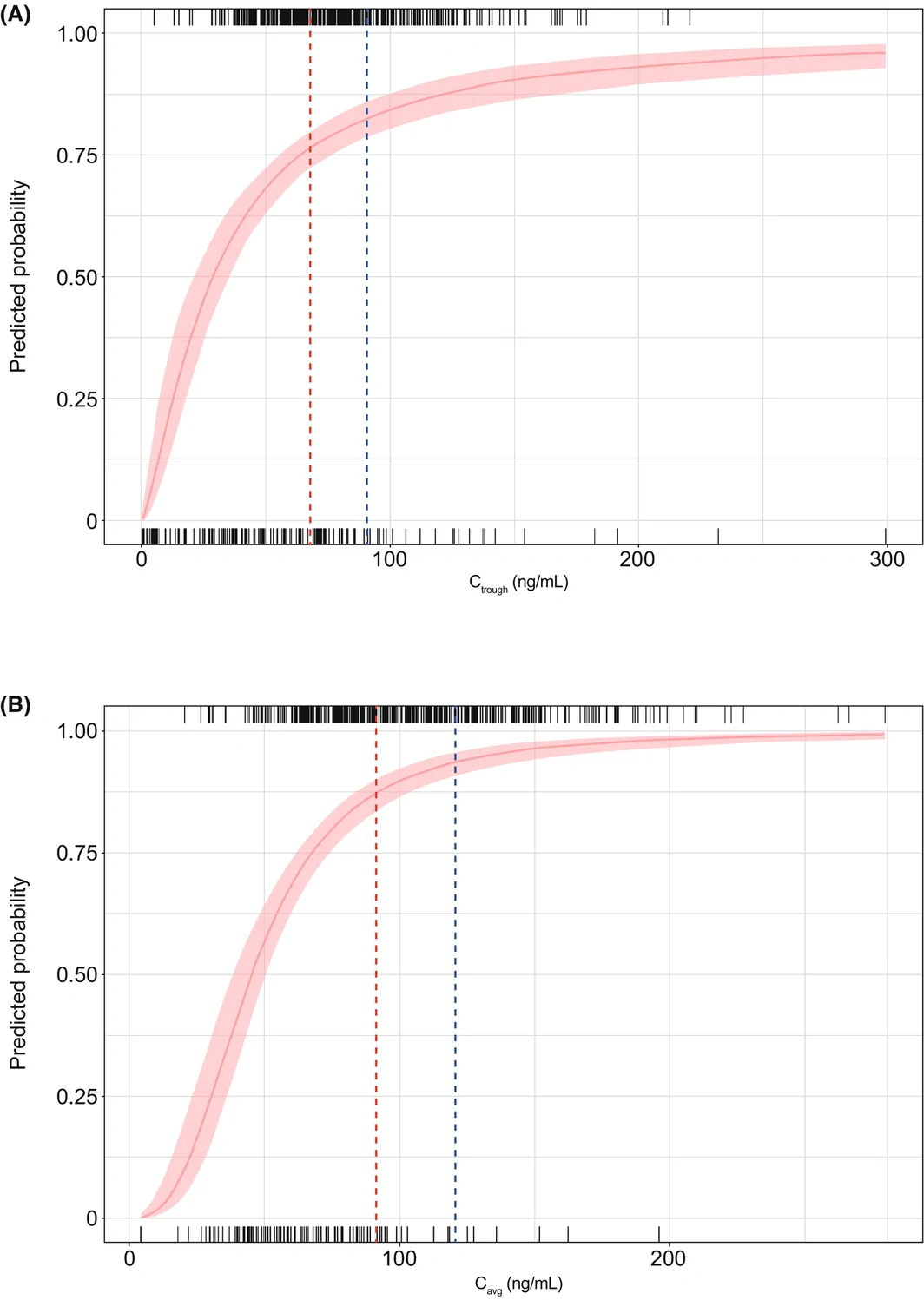
Predicted probability of achieving (A) cumulative MMR with *C*_trough_ (B) cumulative CCyR with *C*_avg_. A total of 573 patients were included in the analysis of MMR, and 554 were included in the CCyR analysis. The red shaded area and solid line are the predicted 95% CI and mean, respectively. The vertical dashed lines are the predicted geometric mean *C*_trough_ and *C*_avg_ for BELA (blue, 500 mg QD starting dose) and geometric mean *C*_trough_ and *C*_avg_ for BFORE and B1871048 (red, 400 mg QD starting dose). *C*_trough_ and *C*_avg_ are presented instead of log(*C*_trough_) and log(*C*_avg_) for ease of interpretation. Black ticks at the top are responders, and at the bottom are non‐responders of actual observed data. *C*_avg_, average concentration at time to initial response; CCyR, complete cytogenetic response; *C*_trough_, predicted trough concentration prior to the event; MMR, major molecular response.

To better illustrate the probability of achieving MMR and CCyR in relation to bosutinib exposures, Figure 1 presents the predicted probability of experiencing MMR and CCyR versus log(*C*_trough_) and log(*C*_avg_), respectively. For MMR at the predicted bosutinib geometric mean *C*_trough_ of 90.7 ng/mL for BELA (500 mg QD starting dose, Table S3) and 67.9 ng/mL for BFORE and B1871048 (400 mg QD starting dose, Table S4), the probability of a response is approximately 83% versus 76%, respectively. For CCyR, at the predicted bosutinib geometric mean *C*_avg_ of 120.8 ng/mL for BELA (500 mg QD starting dose, Table S3) and 91.2 ng/mL for BFORE and B1871048 (400 mg QD starting dose, Table S4), the probability of a response is approximately 94% versus 87%, respectively. At a dose of 400 mg and above, the probability of response is at or near the flat portion of the curve, so additional exposure increases beyond those achieved with the 400 mg dose are projected to have minimal additional efficacy benefit. Furthermore, patients who were in the higher quartiles of bosutinib exposure had a higher probability of response compared with patients in the lower quartiles of exposure (Figure S1).

### 3.4 Exposure–response analysis for key safety endpoints

**FIGURE 2:**
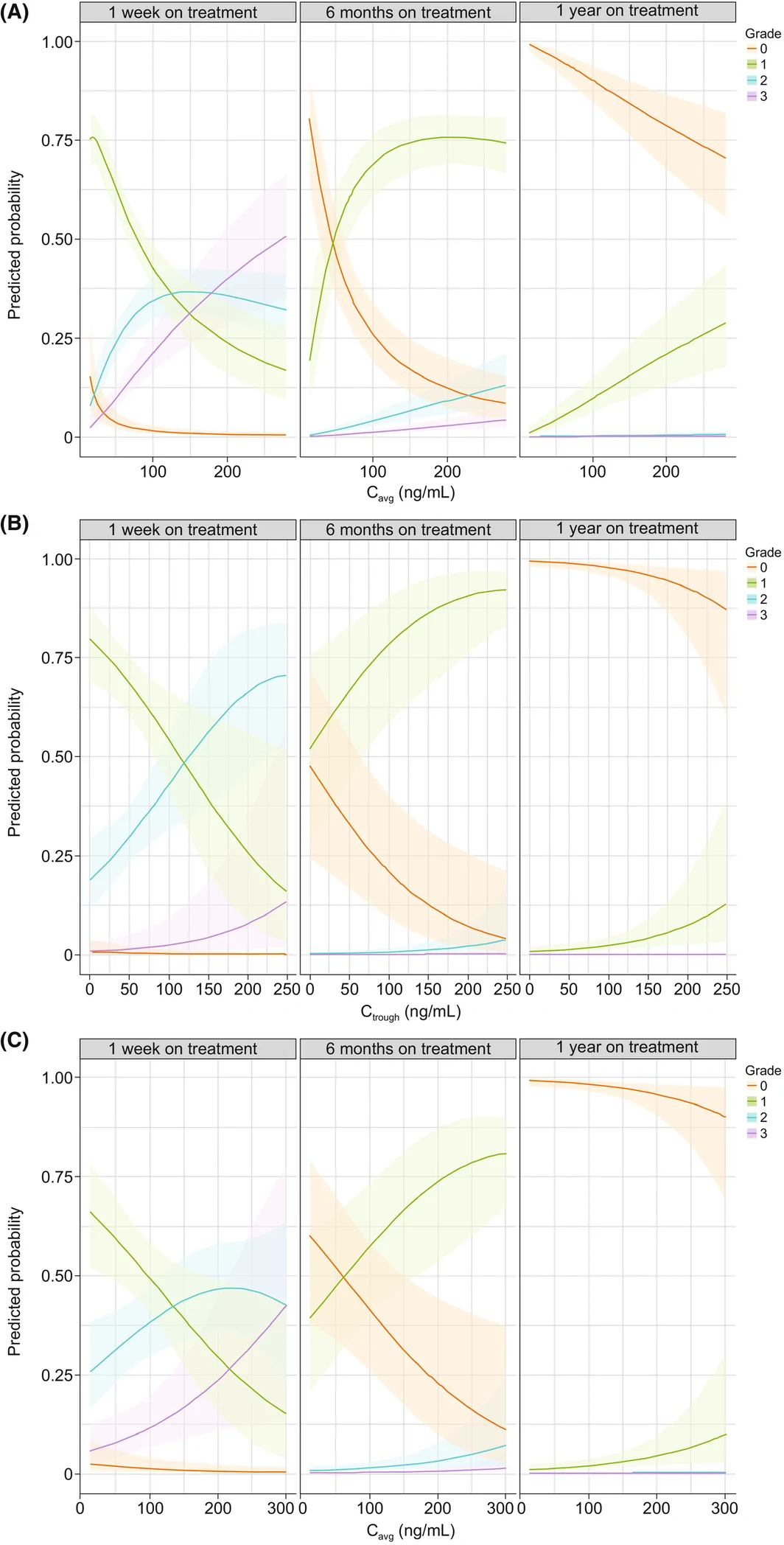
Predicted probability of (A) diarrhea grade with *C*_avg_, (B) nausea grade with *C*_trough_, and (C) vomiting grade with *C*_avg_ by time on treatment. In A: log(*C*_avg_) was the significant exposure for the final model; however, *C*_avg_ is presented for ease of interpretation. The shaded area is the predicted 95% CI, and the solid line is the predicted mean. *C*_avg_, average concentrations prior to event; *C*_trough_, predicted trough concentration prior to the event.

For all safety endpoints, Grade 0 (no event) was the most frequent observed grade. The exception was diarrhea, with 41.3% of patients having Grade 1 and 29.3% having Grade 0 (Table S5). The rates of permanent discontinuations due to AEs in primary analyses were 19.0% in BELA,^7^ 14.2% in BFORE,^8^ and 30.0% in B1871048.^9^ The reason for a higher rate of discontinuation in B1871048 was unclear. The final 3‐year follow‐up of B1871048 demonstrated that most permanent discontinuations due to AEs occurred within the first 6 months of bosutinib treatment.^11^ A significant exposure–response relationship was identified between time‐to‐event and log(*C*_avg_), *C*_trough_, and *C*_avg_ with diarrhea, nausea, and vomiting, respectively (Table S6). No demographic covariates were found to be statistically significant on any of these safety endpoints. To illustrate the probability of incidence of AE grades in relation to bosutinib exposure and time‐to‐event, Figure 2 and Figure S2 present the predicted probability curve with fixed time‐to‐event values or with fixed bosutinib exposure values, respectively. The results show that for three fixed time‐to‐event values (1 week, 6 months, and 1 year), the incidence of any grade AEs is much lower with time on treatment. For the three fixed bosutinib exposure values (minimum, median, and maximum), as exposure increases, the incidence of any grade AEs is much lower with time on treatment. This is plausible since patients who experienced an event earlier either discontinued or, if they remained on study, were more likely to tolerate or optimize treatment with the AE. A significant exposure–response relationship was also identified between *C*_trough_ and incidence of rash and elevated AST (Table S7). In addition, baseline AST was included in the final model for elevated AST. No demographic covariates were found to be statistically significant on any of these safety endpoints. For the incidence of rash and elevated AST, patients have an increased probability of an AE grade with increasing bosutinib exposure (Figure S3).

For safety endpoints of incidence of elevated ALT, neutropenia, and thrombocytopenia, no significant exposure–response relationship was identified using ordinal logistic regression. To further evaluate these safety endpoints, binary logistic regression was performed at different AE grades (i.e., Grade >0, Grade >1, or Grade >2). No significant exposure–response relationship between bosutinib exposure and incidence of any grade AE was observed for elevated ALT and neutropenia. However, a significant exposure–response relationship was identified between thrombocytopenia Grade >2 and *C*_avg28_ and age. In addition, baseline platelets count (BPLTS) was included in the final model. Final model parameter estimates are shown in Table S8 and the final logistic regression equation is presented below.

Final logistic regression equation:

$$\begin{array}{c} \text{logit}\left(p\right)=\log\left(\frac{p}{1-p}\right)=-1.5-0.06\cdot \log\left(\text{BPLTS}\right)\;\left(\times 10^{9}\;\text{cells}/L\right)-0.03\cdot \mathrm{Age}\;\left(\text{years}\right)+0.008\cdot C_{\mathrm{avg}28}\;\left(\mathrm{ng}/\mathrm{mL}\right) \end{array}$$

For the incidence of thrombocytopenia, patients had an increased probability of an AE Grade >2 with increasing bosutinib exposure and a decreased probability of an AE Grade >2 with increasing age (Figure S4).

## 4 DISCUSSION

The exposure–response efficacy analysis demonstrated that cumulative MMR and CCyR were similar in patients who received a bosutinib starting dose of 400 mg QD in the BFORE and B1871048 trials and those who received a starting dose of 500 mg QD in the BELA trial. With regard to the relationships between metrics of bosutinib exposure and key efficacy and safety outcomes, the present analysis found that the log(*C*_trough_) of bosutinib was a significant predictor of cumulative MMR, and the log(*C*_avg_) of bosutinib was a significant predictor of cumulative CCyR.

For safety exposure–response analysis, the risks of experiencing the eight prespecified, clinically relevant AEs were correlated with bosutinib exposure, with higher exposures associated with greater risks of diarrhea, nausea, and vomiting early in the treatment period, and greater risks of experiencing rash, elevated ALT, and elevated AST at any time. The reason for the finding that higher levels of bosutinib exposure were associated with reduced risks of neutropenia and thrombocytopenia is unclear. One hypothetical possibility is that the more rapid suppression of the Ph + cell clone may allow for a more rapid recovery of normal hematopoiesis, minimizing periods of significant myelosuppression. A bosutinib exposure–response relationship was identified with time‐to‐event for diarrhea, nausea, and vomiting, whereas no demographic covariates (e.g., age, sex, Asian vs. non‐Asian) were statistically significant for any of these safety endpoints. Interestingly, as bosutinib exposure increases, the incidence of any AE grade is much lower with time on treatment for diarrhea, nausea, and vomiting. In the final model, a statistically significant relationship between *C*_trough_ and the occurrence of rash and elevated AST was found, whereas no demographic covariates were statistically significant for any of these safety endpoints. Lastly, a statistically significant exposure–response relationship between bosutinib exposure and the probability of Grade >2 severity was observed for thrombocytopenia, whereas no significant exposure–response relationship between bosutinib exposure and incidence for elevated ALT and neutropenia was observed.

In the population PK analysis, age and body weight were statistically significant covariates on bosutinib V_2_, and being Asian was a statistically significant covariate on both bosutinib CL and V_2_. However, these effects had no clinically significant effect on cumulative MMR or CCyR, and no dose adjustments for bosutinib are warranted on the basis of the covariates tested in these analyses. Renal function measured using BCCL was a significant covariate for bosutinib CL. However, changes in bosutinib CL in the mild renal impairment group were minor, supporting the current recommendation of dose adjustment for moderate and severe renal impairment only.^6^, ^12^

According to the NCI ODWG criteria for hepatic impairment (in patients with solid tumors and CML), bosutinib CL did not appear to be altered by mild (B1 and B2) hepatic impairment. However, due to the limitations of the data used in this analysis, caution should be applied when trying to interpret the effect of hepatic impairment on bosutinib exposure. Hence, the current dosing adjustments should be followed based on the results of the dedicated hepatic impairment study (B1871003).^13^

The analyses presented here fulfill a commitment to the European Medicines Agency and the Committee for Medicinal Products for Human Use to update the population PK and exposure–response model with additional PK and safety data from Japanese patients with newly diagnosed CP‐CML and to assess any impact for Asian patients. The relative importance of peak levels and cumulative exposures in determining the safety and efficacy of TKIs used in this clinical setting may differ depending on the specific agent used. Although plasma exposure to an agent would be expected to be lower with a reduced dose, which was the case in the present analysis, the clinical efficacy of the agent may be improved if the number and duration of dose interruptions is minimized over the course of a chronic dosing schedule. Indeed, the importance of a dosing regimen that avoids frequent interruptions has been emphasized by results from recent studies of TKIs other than bosutinib. For example, while several studies of high‐dose imatinib with a rather rigid dosing regimen failed to demonstrate clinical benefit,^14^, ^15^ those that used a flexible dosing schedule or a standard dose wash‐in period did demonstrate benefit.^16^, ^17^

Population PK studies have been used with similar intent for other TKIs in the CML setting to better understand adequacy of dosing and correlation with efficacy and safety.^18^, ^19^, ^20^, ^21^, ^22^, ^23^ However, exposure–response analyses have reported heterogeneous results. For example, an analysis from the IRIS trial of imatinib 400 mg QD showed that patients with a higher steady‐state imatinib *C*_trough_ level had a greater likelihood of achieving CCyR and MMR but also had greater risks of fluid retention, rash, myalgia, and anemia.^18^ This analysis reported that imatinib *C*_trough_ and Sokal risk group were both variables predictive of a patient achieving CCyR, with higher *C*_trough_ levels associated with a greater likelihood of CCyR and higher Sokal risk group associated with a lower likelihood of CCyR. However, the rate of discontinuation due to AEs did not increase across the *C*_trough_ quartiles, which indicates that the incident AEs were manageable. In fact, the highest rate of discontinuation due to AEs was observed in patients with imatinib *C*_trough_ levels within the first quartile, with “unsatisfactory therapeutic effect” being the most common reason for discontinuation.

An exposure–response analysis similar to the present study was conducted for nilotinib using data from the ENESTnd trial.^19^ This study investigated the relationships between PK data and clinical outcomes in two nilotinib dosing groups: 300 mg twice daily (BID) and 400 mg BID. The authors reported no apparent relationship between the *C*_trough_ or AUC_0–24_ of nilotinib and the MMR rate at 12 months. However, patients with higher nilotinib exposures were at greater risk of total bilirubin elevations of any grade and were at greater risk of QT prolongation on electrocardiograms using the Fridericia formula. The authors suggest the relationship between nilotinib exposure and clinical outcomes may not be as obvious as that previously observed with imatinib because nilotinib's greater potency results in the covariates of response and safety being rendered less clinically relevant.

An exposure–response analysis, based on a PK model constructed using data from seven clinical trials of dasatinib, compared the four dasatinib dosing groups included in the Phase 3 trial that contributed the majority of patient data used in the PK model.^20^ The four dosing groups were 140 mg QD, 100 mg QD, 70 mg BID, and 50 mg BID. The weighted, mean steady‐state plasma concentration of dasatinib was associated with achievement of a major cytogenetic response, and dasatinib *C*_trough_ levels were associated with the risk of pleural effusion. This study also found that the likelihood of achieving a major cytogenetic response was significantly associated with the maintenance of uninterrupted dosing. Patients in the 100 mg QD group displayed the lowest steady‐state dasatinib *C*_trough_ levels as well as the highest rate of dose maintenance.

The results from the present analysis should be considered in light of its limitations. This analysis was a retrospective modeling study including data from two clinical trials designed to assess the safety and efficacy of bosutinib compared with imatinib and a single‐arm study of bosutinib alone, none of which were designed for the purpose of generating a PK model used for investigating relationships between metrics of bosutinib exposure and clinical outcomes. The presence of unknown confounders that may have affected the results of the analysis cannot be ruled out. Moreover, while our analysis demonstrates a positive correlation between log(*C*_trough_) and log(*C*_avg_) and MMR and CCyR, respectively, a prospective study is needed to test whether dose modifications based on PK data would improve responses. Given the availability of quantitative polymerase chain reaction as a reliable means of assessing response, it is unlikely that therapeutic drug monitoring will become part of clinical management in the near future. PK analyses have been valuable mostly for population‐based analysis of safety and efficacy correlations, and to optimize dose administration schedules of TKIs.^21^, ^22^

In conclusion, a bosutinib exposure–response relationship with safety and efficacy was observed in adult patients with newly diagnosed CP‐CML. However, the predicted exposures achieved within the recommended dose range (400–500 mg QD) appear to be at or near the plateau for efficacy, which is supported by similar response rates at the 400 and 500 mg dose levels across studies. For safety, patients who received a bosutinib starting dose of 400 mg QD in the BFORE and B1871048 trials had a lower rate of permanent discontinuations due to AEs in some patient populations, while maintaining efficacy—measured by cumulative MMR and cumulative CCyR—at a similar level to those who received a dose of 500 mg QD in the BELA trial. Therefore, we conclude that a bosutinib starting dose of 400 mg QD improved tolerability without compromising efficacy in adult patients with newly diagnosed CP‐CML.

## AUTHOR CONTRIBUTIONS

**May Garrett:** Conceptualization (lead); formal analysis (lead); methodology (lead); writing – original draft (equal); writing – review and editing (equal). **Beverly Knight:** Conceptualization (supporting); formal analysis (supporting); methodology (supporting); writing – original draft (equal); writing – review and editing (equal). **Jorge E Cortes:** Writing – original draft (equal); writing – review and editing (equal). **Michael W Deininger:** Writing – original draft (equal); writing – review and editing (equal).

## FUNDING INFORMATION

This study was sponsored by Pfizer.

## CONFLICT OF INTEREST STATEMENT

**May Garrett** is an employee of Pfizer. **Beverly Knight** was an employee of Pfizer when the included studies were conducted and is an employee of Janssen at the time of manuscript submission. **Jorge E Cortes** served as a consultant for Amphivena Therapeutics, Astellas Pharma, Bio‐Path Holdings Inc, BiolineRx, Bristol Myers Squibb, Daiichi Sankyo, Jazz Pharmaceuticals, Novartis, Pfizer, and Takeda; and received research funding from Astellas Pharma, Bristol Myers Squibb, Daiichi Sankyo, Immunogen, Jazz Pharmaceuticals, Merus, Novartis, Pfizer, Sun Pharma, Takeda, Tolero Pharmaceuticals, and Tovagene. **Michael W Deininger** served as a consultant for Ariad, Blueprint Medicine, Bristol Myers Squibb, CTI Biopharma, Cogent, Galena Biopharma, Incyte, Novartis, and Pfizer; received research funding from Pfizer.

## ETHICS STATEMENT

The study was approved by institutional review boards/independent ethics committees at each center. The study was conducted in accordance with all local legal and regulatory requirements, and in accordance with the International Conference on Harmonization Guideline for Good Clinical Practice and the Declaration of Helsinki.

## PATIENT CONSENT STATEMENT

All patients provided written informed consent.

## CLINICAL TRIAL REGISTRATION

ClinicalTrials.gov identifiers: NCT00574873; NCT02130557; NCT03128411.

## Supporting information


Data S1:

## Data Availability

Upon request, and subject to review, Pfizer will provide the data that support the findings of this study. Subject to certain criteria, conditions and exceptions, Pfizer may also provide access to the related individual de‐identified participant data. See https://www.pfizer.com/science/clinical‐trials/trial‐data‐and‐results for more information.
