# Supplementary material for: Population modeling of bosutinib exposure‐response in patients with newly diagnosed chronic phase chronic myeloid leukemia
Source: Cancer Med. 2023 Aug 8;12(17):17981–92. doi: 10.1002/cam4.6439 (PMC10524044; doi:10.1002/cam4.6439)
Supplement: Supplementary file 1 — Data S1: [file CAM4-12-17981-s001.pdf]

SUPPLEMENTARY MATERIAL

Population Modeling of Bosutinib Exposure-Response in Patients with Newly Diagnosed Chronic Phase Chronic Myeloid Leukemia

May Garrett, BS[1], Beverly Knight, PhD[1]*, Jorge E. Cortes, MD[2], Michael W. Deininger, MD, PhD[3]

[1]Pfizer Oncology, San Diego, CA, USA;
[2]Georgia Cancer Center, Augusta, GA, USA;
[3]Versiti Blood Research Institute, Milwaukee, WI, USA
*Affiliation at the time when included studies were conducted

Figure S1. Risk score of (A) MMR and (B) CCyR. A total of 573 patients were included in the analysis of MMR, and 554 were included in the CCyR analysis.
C_{avg}: average concentration at time to initial response; CCyR: cumulative complete cytogenetic response; C_{trough}: predicted trough concentration prior to the event; MMR: cumulative major molecular response; (-): no response; (+): response

A

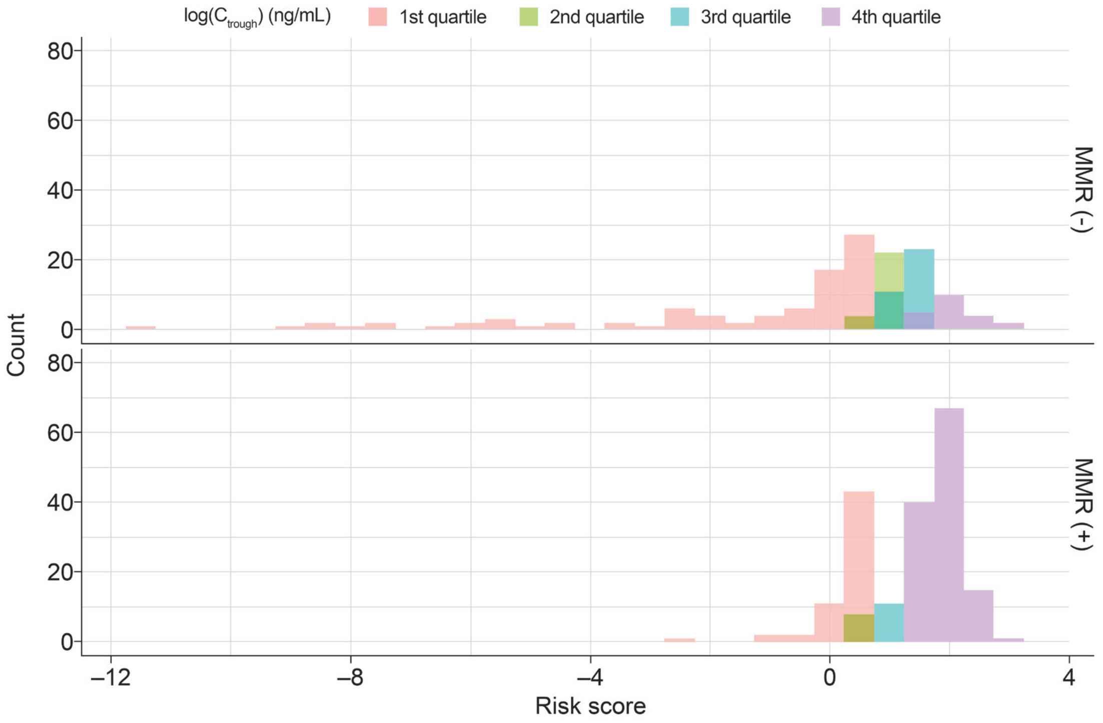

B

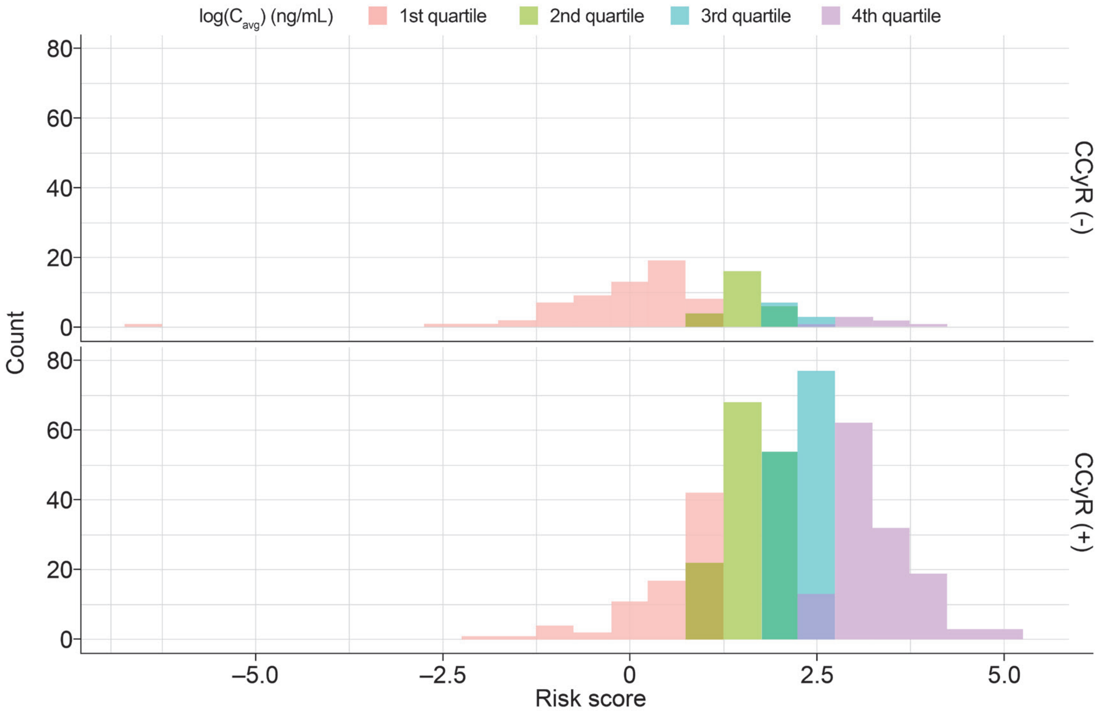

Figure S2. Predicted probability of (A) diarrhea grade by minimum, median, and maximum C_{avg}, (B) nausea grade with by minimum, median, and maximum C_{trough}, and (C) vomiting grade by minimum, median, and maximum C_{avg} with time on treatment. In A: log(C_{avg}) was the significant exposure for the final model; however, C_{avg} is presented for ease of interpretation. The shaded area is the predicted 95% CI, and the solid line is the predicted mean.

C_{avg}: average concentrations prior to event; C_{trough}: predicted trough concentration prior to the event

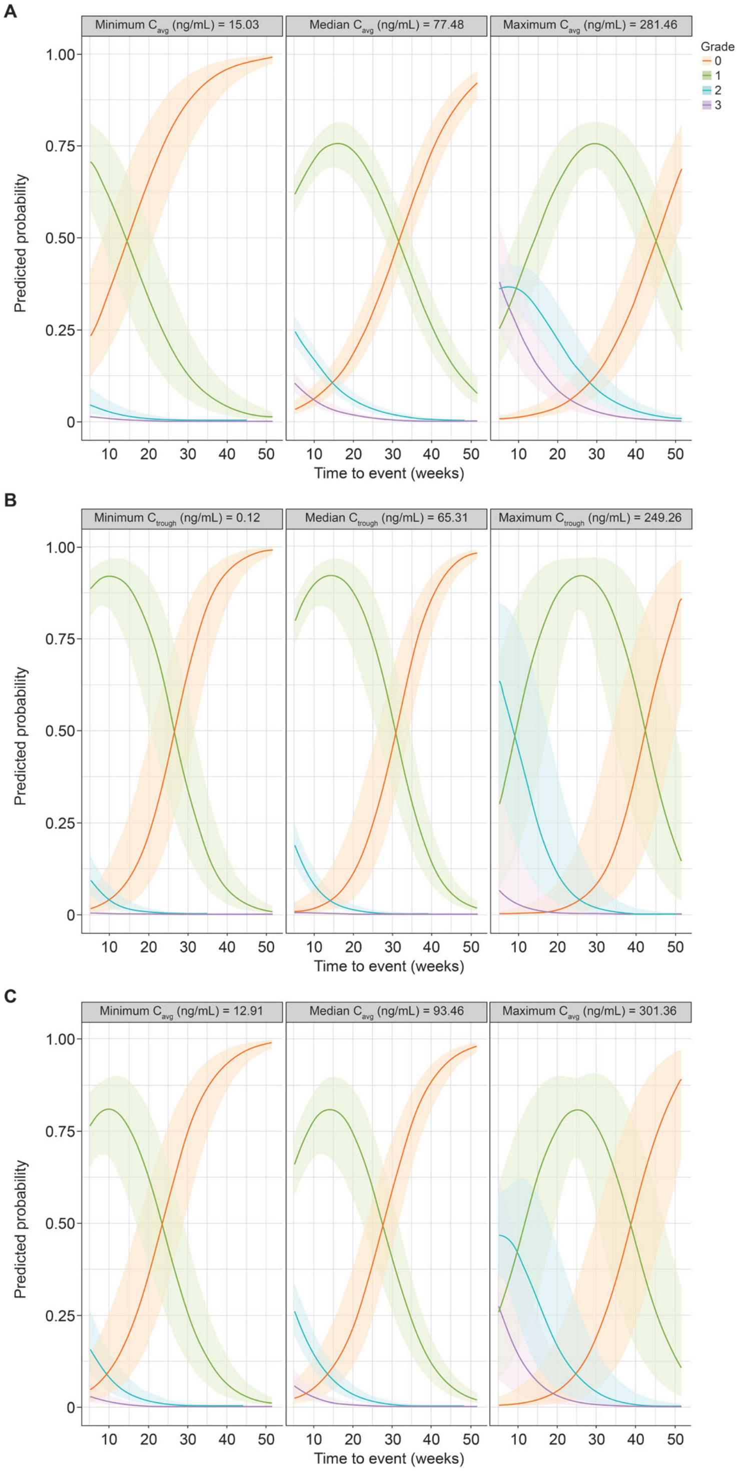

Figure S3. Predicted probability of (A) rash grade with C_{trough} and (B) elevated AST grade with C_{trough}. The shaded area is the predicted 95% CI, and the solid line is the predicted mean.
AST: aspartate aminotransferase; C_{trough}: predicted trough concentration prior to event

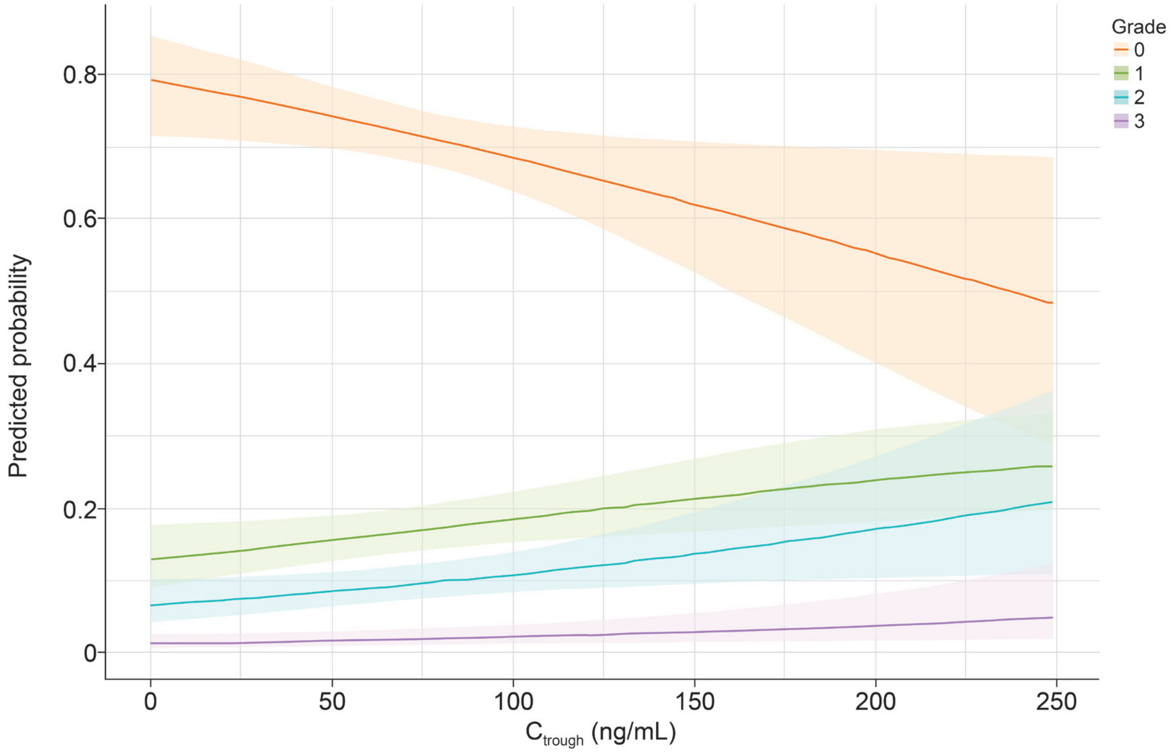

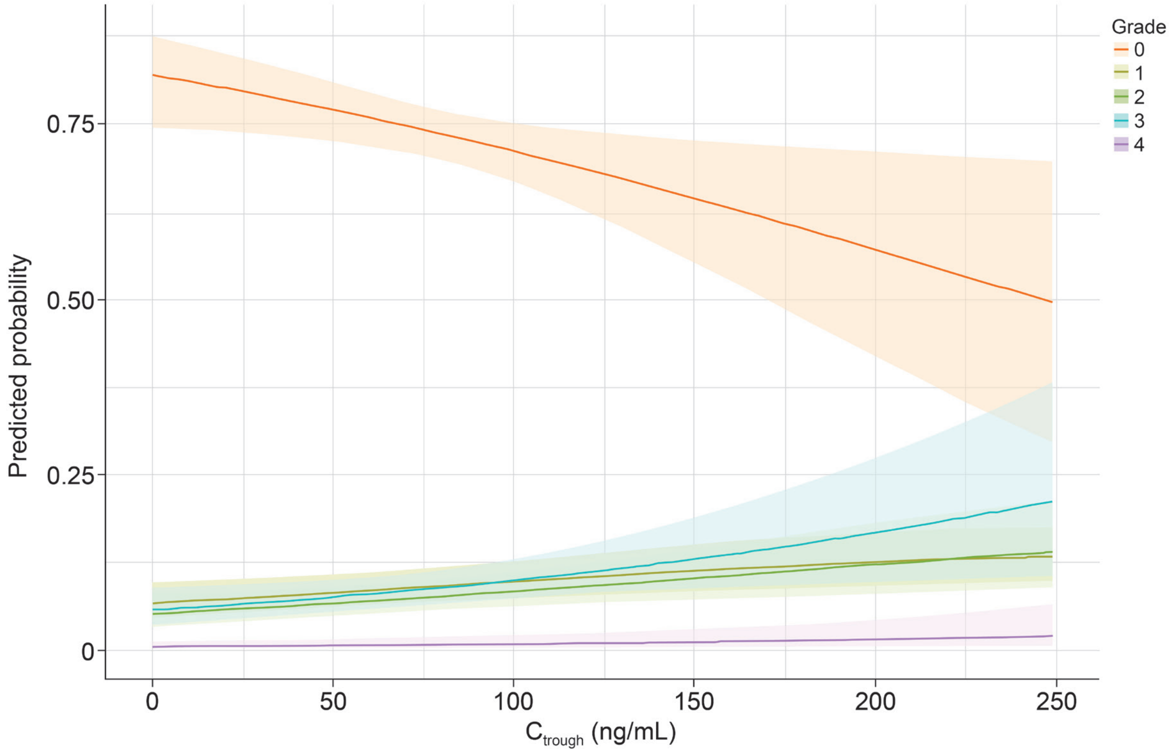

Figure S4. Predicted probability of thrombocytopenia grade >2 with (A) C_{avg28} by quartiles of age and (B) age by minimum, median, and maximum C_{avg28}. The shaded area is the predicted 95% CI, the solid line is the predicted mean. In A: at the fixed analysis population median C_{avg28} of 91.8 ng/mL (blue dashed line), the predicted probability of thrombocytopenia grade >2 is approximately 11%, 8%, and 7% at the analysis population 25th, 50th, and 75th quartiles of age. In B: at the analysis population median age of 50 years (blue dashed line), the predicted probability of thrombocytopenia grade >2 is approximately 5%, 8%, and 27% at the minimum, median, and maximum C_{avg28}.

C_{avg28}: average concentration calculated as the ratio of cAUC28 over 28 days.

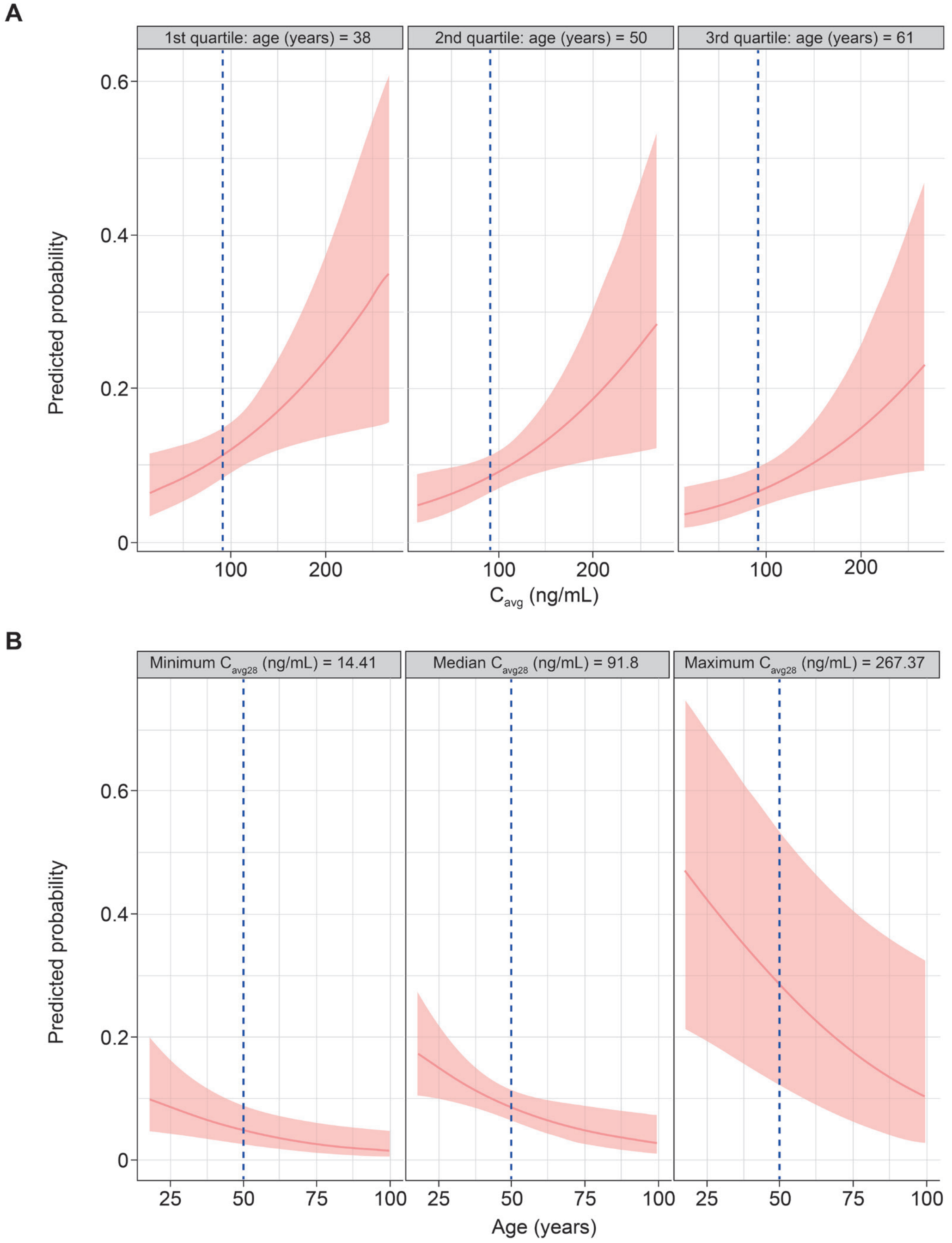

Table S1. Metrics of bosutinib exposure in PK-evaluable patients

Parameter	BELA ($N = 247$)	BFORE ($N = 266$)	B1871048 ($N = 60$)
**Endpoint: MMR**[a]			
cAUC, µg·h/mL			
Median (range)	653.5 (25.5–9236.1)	496.9 (12.1–5809.3)	365.9 (24.6–3915.0)
Mean (SD)	1154.2 (1335.1)	782.8 (840.6)	519.6 (686.3)
C_{avg}, ng/mL			
Median (range)	108.5 (4.4–296.0)	84.2 (11.7–226.2)	90.5 (19.2–191.3)
Mean (SD)	114.3 (48.1)	87.7 (33.3)	90.8 (39.9)
C_{trough}, ng/mL			
Median (range)	77.7 (0.1–299.3)	62.9 (0.01–211.9)	67.9 (5.2–176.6)
Mean (SD)	82.7 (44.3)	63.3 (33.1)	72.4 (31.8)
$cAUC_{28}$, µg·h/mL			
Median (range)	69.2 (18.5–173.4)	52.1 (9.3–129.6)	59.2 (22.0–125.6)
Mean (SD)	73.9 (28.6)	54.7 (18.8)	58.7 (22.5)
C_{avg28}, ng/mL			
Median (range)	106.8 (28.6–267.5)	80.4 (14.4–200.1)	91.4 (33.9–193.8)
Mean (SD)	114.0 (44.1)	84.4 (29.0)	90.5 (34.8)
$C_{trough28}$, ng/mL			
Median (range)	85.6 (0.6–247.5)	63.8 (0.4–191.4)	75.7 (2.0–174.9)
Mean (SD)	90.3 (43.5)	67.3 (29.0)	80.5 (30.8)
**Endpoint: CCyR (Ph+ patients only)**[b]			
cAUC, µg·h/mL			
Median (range)	279.1 (25.5–4671.6)	256.0 (15.7–1553.2)	210.7 (24.6–602.9)
Mean (SD)	360.0 (370.0)	321.2 (237.6)	236.5 (138.9)
C_{avg}, ng/mL			
Median (range)	108.1 (4.4–279.6)	79.0 (18.1–188.2)	87.2 (20.6–191.3)
Mean (SD)	112.3 (45.5)	84.1 (31.3)	86.4 (37.7)
C_{trough}, ng/mL			
Median (range)	80.4 (0.2–249.4)	61.3 (0.01–211.1)	66.8 (0.1–154.6)
Mean (SD)	86.6 (40.8)	63.6 (31.4)	69.7 (31.3)
$cAUC_{28}$, µg·h/mL			
Median (range)	69.2 (18.5–173.4)	52.0 (9.3–129.6)	59.2 (22.0–125.6)
Mean (SD)	73.9 (28.6)	54.7 (18.8)	58.7 (22.5)
C_{avg28}, ng/mL			
Median (range)	106.8 (28.6–267.5)	80.2 (14.4–200.1)	91.4 (33.9–193.8)
Mean (SD)	114.0 (44.1)	84.5 (29.0)	90.5 (34.8)
$C_{trough28}$, ng/mL			
Median (range)	85.6 (0.6–247.5)	63.7 (0.4–191.4)	75.7 (2.0–174.9)
Mean (SD)	90.3 (43.5)	67.0 (29.1)	80.5 (30.8)

[a]A total of 573 patients were included in the exposure-response analysis of cumulative MMR.
[b]Only Ph+ patients were included in the analysis of cumulative CCyR ($N = 554$).
AUC: area under the concentration-time curve; cAUC: cumulative AUC up to time to initial response; AUC_{28}: cumulative AUC up to Day 28; CCyR: complete cytogenetic response; C_{trough}: trough concentration prior to initial response; $C_{trough28}$: trough concentration on Day 28; C_{avg}: average concentration calculated as the ratio of cAUC over the respective time frame; C_{avg28}: average concentration calculated as the ratio of cAUC28 over the respective time frame; MMR: major molecular response

Table S2. Final logistic regression estimates for key efficacy endpoints

Variables	Estimate (95% CI)	z value	Probability>|z|[a]
**MMR**			
Intercept	-4.5 (-6.1, -3.1)	-5.9	<0.0001
log(C_{trough}), ng/mL	1.3 (1.0, 1.7)	7.2	<0.0001
Odds ratio: log(C_{trough}), ng/mL	3.8 (2.7, 5.7)	NA	NA
ΔD	111.2	NA	NA
AIC	582.6	NA	NA
df	1	NA	NA
1-p-value	<0.0001	NA	NA
Log-likelihood	-289.3	NA	NA
**CCyR**			
Intercept	-10.5 (-13.3, -7.9)	-7.7	<0.0001
log(C_{avg}), ng/mL	2.7 (2.1, 3.4)	8.6	<0.0001
Odds ratio: log(C_{avg}), ng/mL	15.6 (8.5, 30.0)	NA	NA
ΔD	102.6	NA	NA
AIC	436.5	NA	NA
df	1	NA	NA
1-p-value	<0.0001	NA	NA
Log-likelihood	-216.2	NA	NA

[a]Probability of observing a value greater than the z value.

AIC: Akaike information criterion; C_{avg}: average concentration calculated as the ratio of cAUC over the respective time frame; C_{trough}: trough concentration at time to initial response; CCyR: cumulative complete cytogenetic response; ΔD: deviance between the null and residual; df: degrees of freedom; MMR: major molecular response; NA: not applicable; |z|: tail area in a 2-tail test

Table S3. Summary of simulated PK parameters in patients with CML from BELA

Parameter	Median (range)	Mean (SD)	Geometric mean (CV%)
C_{max}, ng/mL			
Day 1	94.2 (22.3–231.9)	98.0 (37.9)	90.6 (38.6)
Day 28	174.1 (36.6–446.4)	183.6 (70.0)	170.6 (38.2)
Day 56	174.2 (36.6–447.3)	184.0 (70.5)	170.9 (38.3)
C_{trough}, ng/mL			
Day 1	–	–	–
Day 28	93.8 (15.0–247.3)	99.0 (41.7)	90.7 (42.2)
Day 56	94.4 (15.0–249.3)	99.5 (42.3)	91.0 (42.5)
C_{avg}, ng/mL			
Day 1	62.8 (17.8–129.6)	63.8 (19.8)	60.5 (31.0)
Day 28	125.0 (28.6–296.6)	128.6 (45.2)	120.8 (35.1)
Day 56	130.3 (29.0–315.9)	135.0 (49.2)	126.2 (36.4)
AUCτ, μg·h/mL			
Day 1	1.5 (0.4–3.1)	1.5 (0.5)	1.5 (31.0)
Day 28	3.3 (0.7–8.0)	3.4 (1.3)	3.2 (37.5)
Day 56	3.3 (0.7–8.1)	3.4 (1.3)	3.2 (37.8)
cAUC, μg·h/mL			
Day 1	1.5 (0.4–3.1)	1.5 (0.5)	1.5 (31.0)
Day 28	84.0 (19.2–199.3)	86.5 (30.4)	81.2 (35.1)
Day 56	175.1 (39.0–424.6)	181.4 (66.1)	169.6 (36.4)

A total of 248 patients were simulated at a fixed continuous dosing regimen of 500 mg QD (starting dose for BELA).
AUC: area under the concentration-time curve; AUCτ: AUC from time 0 to 24 hours; cAUC: cumulative AUC up to time to initial response; C_{avg}: average concentration calculated as the ratio of cAUC over the respective time frame; C_{trough}: trough concentration prior to initial response; C_{max}: maximum concentration; CML: chronic myeloid leukemia; CV%: coefficient of variation; PK: pharmacokinetic

Table S4. Summary of simulated PK parameters in patients with CML from BFORE and B1871048

Parameter	Median (range)	Mean (SD)	Geometric mean (CV%)
C_{max}, ng/mL			
Day 1	67.7 (25.6–166.1)	70.6 (17.9)	68.3 (25.4)
Day 28	127.1 (29.3–313.5)	133.5 (41.5)	127.4 (31.1)
Day 56	127.2 (29.3–322.3)	133.8 (41.8)	127.5 (31.3)
C_{trough}, ng/mL			
Day 1	–	–	–
Day 28	68.3 (4.7–202.3)	73.4 (29.0)	67.9 (39.5)
Day 56	68.3 (4.7–211.3)	73.6 (29.3)	68.1 (39.9)
C_{avg}, ng/mL			
Day 1	46.6 (12.2–86.0)	48.0 (11.2)	46.7 (23.2)
Day 28	91.6 (14.4–210.5)	95.8 (29.8)	91.2 (31.1)
Day 56	95.3 (14.5–238.4)	100.3 (32.6)	95.2 (32.5)
AUCτ, µg·h/mL			
Day 1	1.1 (0.3–2.1)	1.2 (0.3)	1.1 (23.2)
Day 28	2.4 (0.4–6.2)	2.5 (0.9)	2.4 (33.7)
Day 56	2.4 (0.4–6.4)	2.5 (0.9)	2.4 (34.0)
cAUC, µg·h/mL			
Day 1	1.1 (0.3–2.1)	1.2 (0.3)	1.1 (23.2)
Day 28	61.6 (9.7–141.5)	64.4 (20.0)	61.3 (31.1)
Day 56	128.1 (19.5–320.4)	134.8 (43.9)	127.9 (32.5)

A total 326 patients were simulated at a fixed continuous dosing regimen of 400 mg QD (starting dose for BFORE and B1871048).

AUC: area under the concentration-time curve; AUCτ: AUC from time 0 to 24 hours; cAUC: cumulative AUC up to time to initial response; C_{avg}: average concentration calculated as the ratio of cAUC over the respective time frame; C_{trough}: trough concentration prior to initial response; C_{max}: maximum concentration; CML: chronic myeloid leukemia; CV%: coefficient of variation; PK: pharmacokinetic

Table S5. Summary of safety endpoints by adverse event grades

Adverse event, *n* (%)	Grade	BELA (*N* = 248)	BFORE (*N* = 266)	B1871048 (*N* = 60)	Total (*N* = 574)
Diarrhea	0	79 (31.9)	81 (30.5)	8 (13.3)	168 (29.3)
	1	99 (39.9)	114 (42.9)	24 (40.0)	237 (41.3)
	2	44 (17.7)	50 (18.8)	19 (31.7)	113 (19.7)
	3	26 (10.5)	21 (7.9)	9 (15.0)	56 (9.8)
	4	0 (0)	0 (0)	0 (0)	0 (0)
Rash	0	184 (74.2)	192 (72.2)	33 (55.0)	409 (71.3)
	1	37 (14.9)	48 (18.0)	13 (21.7)	98 (17.1)
	2	22 (8.9)	22 (8.3)	12 (20.0)	56 (9.8)
	3	5 (2.0)	4 (1.5)	2 (3.3)	11 (1.9)
	4	0 (0)	0 (0)	0 (0)	0 (0)
Nausea	0	173 (69.8)	174 (65.4)	43 (71.7)	390 (67.9)
	1	51 (20.6)	74 (27.8)	11 (18.3)	136 (23.7)
	2	22 (8.9)	18 (6.8)	6 (10.0)	46 (8.0)
	3	2 (0.8)	0 (0)	0 (0)	2 (0.3)
	4	0 (0)	0 (0)	0 (0)	0 (0)
Vomiting	0	172 (69.4)	218 (82.0)	45 (75.0)	435 (75.8)
	1	38 (15.3)	36 (13.5)	11 (18.3)	85 (14.8)
	2	31 (12.5)	9 (3.4)	3 (5.0)	43 (7.5)
	3	7 (2.8)	3 (1.1)	1 (1.7)	11 (1.9)
	4	0 (0)	0 (0)	0 (0)	0 (0)
Elevated ALT	0	171 (69.0)	186 (69.9)	27 (45.0)	384 (66.9)
	1	12 (4.8)	9 (3.4)	8 (13.3)	29 (5.1)
	2	22 (8.9)	20 (7.5)	5 (8.3)	47 (8.2)
	3	35 (14.1)	45 (16.9)	18 (30.0)	98 (17.1)
	4	8 (3.2)	6 (2.3)	2 (3.3)	16 (2.8)
Elevated AST	0	185 (74.6)	207 (77.8)	32 (53.3)	424 (73.9)
	1	21 (8.5)	20 (7.5)	10 (16.7)	51 (8.9)
	2	24 (9.7)	13 (4.9)	7 (11.7)	44 (7.7)
	3	15 (6.0)	25 (9.4)	11 (18.3)	51 (8.9)
	4	3 (1.2)	1 (0.4)	0 (0)	4 (0.7)
Thrombocytopenia	0	183 (73.8)	203 (76.3)	55 (91.7)	441 (76.8)
	1	20 (8.1)	27 (10.2)	2 (3.3)	49 (8.5)
	2	15 (6.0)	12 (4.5)	1 (1.7)	28 (4.9)
	3	20 (8.1)	16 (6.0)	2 (3.3)	38 (6.6)
	4	10 (4.0)	8 (3.0)	0 (0)	18 (3.1)
Neutropenia	0	216 (87.1)	238 (89.5)	50 (83.3)	504 (87.8)
	1	5 (2.0)	1 (0.4)	1 (1.7)	7 (1.2)
	2	8 (3.2)	10 (3.8)	2 (3.3)	20 (3.5)
	3	11 (4.4)	13 (4.9)	7 (11.7)	31 (5.4)
	4	8 (3.2)	4 (1.5)	0 (0)	12 (2.1)

Two patients from the BFORE trial were excluded from the safety analysis due to missing pharmacokinetic data.
AE: adverse event; ALT: alanine transaminase; AST: aspartate transaminase

Table S6. Final ordinal logistic regression estimates with time-to-event parameter for key safety endpoints

Variables	Estimate (95% CI)	z value	Probability>|z|[a]
Diarrhea (n = 574)			
Intercept: Grade 0 to 1	1.7 (-0.2, 3.6)	1.7	0.08
Intercept: Grade 1 to 2	5.6 (3.7, 7.5)	5.7	<0.0001
Intercept: Grade 2 to 3	7.2 (5.2, 9.1)	7.1	<0.0001
log(C_{avg}), ng/mL	1.3 (0.8, 1.7)	5.7	<0.0001
Time to event	-6.5 (-7.3, 5.7)	-15.4	<0.0001
Odds ratio: log(C_{avg}), ng/mL	3.7 (2.3, 5.7)		
Odds ratio: time to event	0.002 (0.0007, 0.003)		
Nausea (n = 574)			
Intercept: Grade 0 to 1	-5.2 (-6.9, 3.4)	-5.9	<0.0001
Intercept: Grade 1 to 2	1.2 (0.6, 1.8)	4.1	0.0001
Intercept: Grade 2 to 3	4.7 (3.2, 6.2)	6.2	<0.0001
C_{trough}, ng/mL	0.01 (0.004, 0.02)	2.8	0.006
Time to event	-10.2 (-12.2, 8.1)	-9.8	<0.0001
Odds ratio: C_{trough}, ng/mL	1.0 (1.0, 1.0)		
Odds ratio: time to event	0 (0.0, 0.0003)		
Vomiting (n = 574)			
Intercept: Grade 0 to 1	-3.8 (-5.1, 2.4)	-5.4	<0.0001
Intercept: Grade 1 to 2	0.7 (-0.003, 1.5)	2.0	0.05
Intercept: Grade 2 to 3	2.7 (1.8, 3.7)	5.9	<0.0001
C_{avg}, ng/mL	0.009 (0.002, 0.02)	2.5	0.01
Time to event	-8.6 (-10.1, 7.1)	-11.1	<0.0001
Odds ratio: C_{avg}, ng/mL	1.0 (1.0, 1.0)		
Odds ratio: time to event	2×10^{-04} (0.0000, 0.0009)		

[a]Probability of observing a value greater than the z value.

C_{avg}: average concentration prior to event; C_{trough}: predicted trough concentration prior to event; N: total number of patients with recorded safety endpoint; n: number of patients included in final model dataset; |z|: tail area in a 2-tail test

Table S7. Final ordinal logistic regression estimates without time-to-event parameter for key safety endpoints

Variables	Estimate (95% CI)	z value	Probability>|z|[a]
Rash (*n* = 574)			
Intercept: Grade 0 to 1	1.3 (0.9, 1.8)	6.3	<0.0001
Intercept: Grade 1 to 2	2.5 (2.0, 2.9)	10.4	<0.0001
Intercept: Grade 2 to 3	4.4 (3.7, 5.1)	12.0	<0.0001
C_{trough}, ng/mL	0.006 (8×10^{-04}, 0.01)	2.3	0.02
Odds ratio: C_{trough}, ng/mL	1.0 (1.0, 1.0)		
Elevated AST (*n* = 569)			
Intercept: Grade 0 to 1	3.6 (1.8, 5.3)	4.0	0.0001
Intercept: Grade 1 to 2	4.1 (2.4, 5.9)	4.6	<0.0001
Intercept: Grade 2 to 3	4.8 (3.0, 6.6)	5.3	<0.0001
Intercept: Grade 3 to 4	7.5 (5.5, 9.5)	7.3	<0.0001
C_{trough}, ng/mL	0.006 (0.001, 0.01)	2.5	0.01
log(BAST), U/L	0.6 (0.1, 1.2)	2.4	0.02
Odds ratio: C_{trough}, ng/mL	1.0 (1.0, 1.0)		
Odds ratio: log(BAST), U/L	1.9 (1.1, 3.2)		

[a]Probability of observing a value greater than the z value.

BAST: baseline aspartate aminotransferase; C_{trough}: predicted trough concentration prior to event; N: total number of patients with recorded safety endpoint; n: number of patients included in final model dataset; |z|: tail area in a 2-tail test

Table S8. Final logistic regression estimates for thrombocytopenia grade >2

Variables	Estimate (95% CI)	z value	Probability>|z|[a]
Intercept	-1.5 (-4.4, 1.3)	-1.0	0.3
C_{avg28}, ng/mL	0.008 (0.002, 0.01)	2.5	0.01
log(BPLTS), $\times 10^9$ cells/L	-0.06 (-0.5, 0.4)	-0.3	0.8
Age, years	-0.03 (-0.04, 0.007)	-2.6	0.009
Odds ratio: C_{avg28}, ng/mL	1.0 (1.0, 1.0)		
Odds ratio: log(BPLTS), $\times 10^9$ cells/L	0.9 (0.6, 1.5)		
Odds ratio: age, years	1.0 (1.0, 1.0)		
ΔD	12.7		
AIC	360.5		
df	3		
1-p-Value	0.005		
Log-likelihood	-176.2		

[a]Probability of observing a value greater than the z value. 565 out of 574 patients were included in the safety analysis for thrombocytopenia.

BAST: baseline aspartate aminotransferase; BPLTS: baseline absolute platelet counts; C_{avg28}: average concentration calculated as the ratio of cumulative area under-the-curve up to Day 28 of treatment over 28 days; |z|: tail area in a 2-tail test
